# First Report of a Neotropical Agaric (*Lepiota spiculata*, Agaricales, Basidiomycota) Containing Lethal α-Amanitin at Toxicologically Relevant Levels

**DOI:** 10.3389/fmicb.2020.01833

**Published:** 2020-08-11

**Authors:** Claudio Angelini, Alfredo Vizzini, Alfredo Justo, Alberto Bizzi, Paolo Davoli, Ertuğrul Kaya

**Affiliations:** ^1^National Botanical Garden of Santo Domingo (JBSD), Santo Domingo, Dominican Republic; ^2^Dipartimento di Scienze della Vita e Biologia dei Sistemi, Università di Torino, Turin, Italy; ^3^New Brunswick Museum, Saint John, NB, Canada; ^4^Independent Researcher, Montecchio Maggiore, Italy; ^5^Independent Researcher, Vignola, Italy; ^6^Department of Pharmacology, Faculty of Medicine, Duzce University, Duzce, Turkey

**Keywords:** Agaricomycetes, Agaricaceae, *Lepiota*, molecular and chemical analyses, taxonomy, toxins, poisoning risk

## Abstract

A recent collection of *Lepiota spiculata* from the Dominican Republic is presented here. Macro- and micromorphological features of *L. spiculata* are described in detail, and its evolutionary (phylogenetic) position within *Lepiota* sect. *Ovisporae*, in the subincarnata/brunneoincarnata clade, is assessed on the basis of a combined nrLSU + nrITS + *rpb2* + *tef1* analysis. Additionally, high levels of deadly amatoxins were detected and quantified in *L. spiculata* for the first time by HPLC analysis; in particular, α-amanitin was found at concentrations up to approximately 4 mg/g dry weight, which render *L. spiculata* a potentially lethal mushroom, if ingested.

## Introduction

**FIGURE 1:**
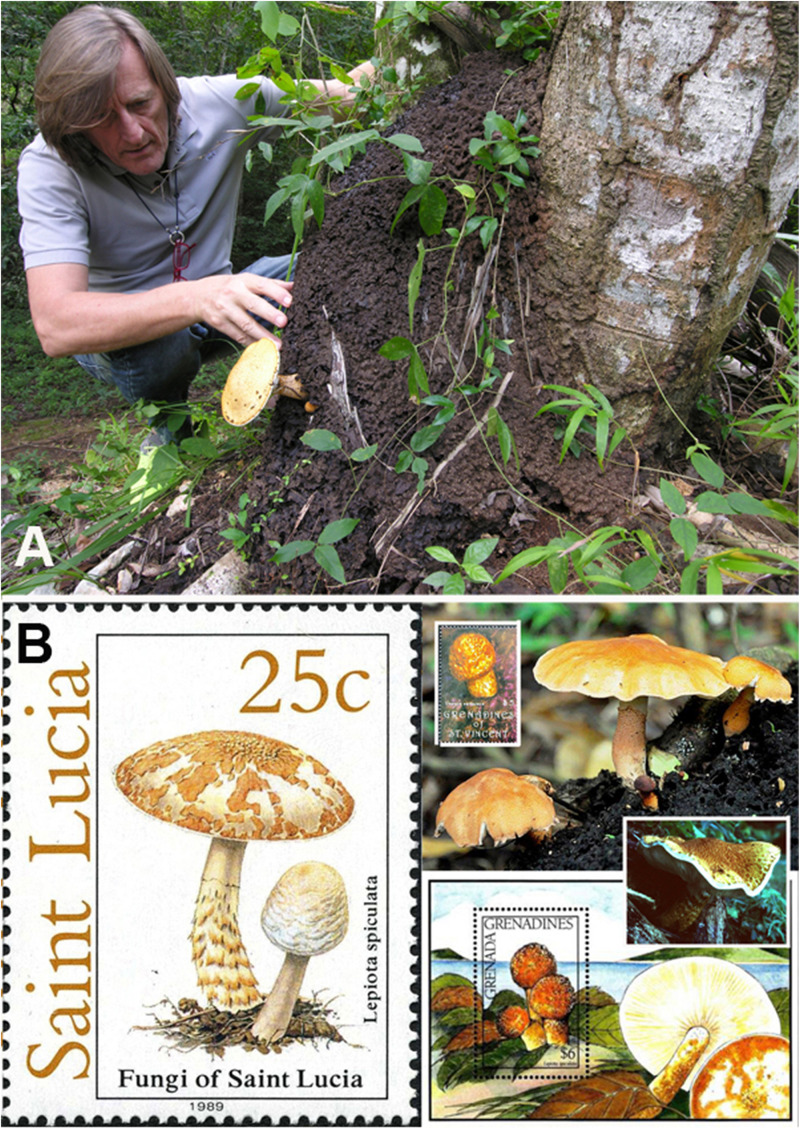
*Lepiota spiculata.* **(A)** Basidiomes in habitat growing on a termite nest (photo by C. Angelini). **(B)** Representations on stamps and websites. Person in image is the first author.

*Lepiota spiculata* was originally described by Pegler (1983) from the Lesser Antilles (Martinique Island), and since then it has been reported so far only from Mexico (Singer and Garcia, 1989). This would suggest that it is an uncommon or even rare mushroom. Despite scarce official reports, however, postage stamps from neotropical countries depicting *L. spiculata* (Saint Lucia, Saint Vincent and Grenadines) (Figure 1B) and photo vouchers from accredited websites (Baroni, 1998) from Puerto Rico (Figure 1B) and on social networks (from Trinidad and Tobago – J. Wong Sang, *personal communication*) (Figure 1B) indicate that this species might be more frequent in the Neotropics than we think. Vellinga (2004a) suggests that it is a neotropical species. The report of *L. spiculata* in China (Mao, 2000; Sysouphanthong et al., 2011) is dubious as the illustration and macroscopic and microscopic data presented in the book “The Macrofungi in China” (Mao, 2000) suggest that this collection likely represents a separate, unknown lepiotoid species (in agreement with Vellinga EC – *personal communication*). All reports of *L. spiculata* are on abandoned termite nests (Pegler, 1983; Singer and Garcia, 1989; Vellinga, 2004a), usually attached to the base of deciduous trees. Our own recent collection from the Dominican Republic described here was growing on an abandoned and decayed nest of the termite *Nasutitermes corniger* Motschulsky (= *N. costalis* Holmgren), locally referred as “*comején*” (Figure 1A).

This collection represents the first official report of *L. spiculata* from the Greater Antilles (Hispaniola Island, Dominican Republic) and allowed us to document its morphology in different growth stages (from primordium to mature basidiome) and to clarify its evolutionary (phylogenetic) position by analyzing two molecular datasets: a combined nrLSU (28S) + nrITS + *rpb2* + *tef1* dataset and a nrITS-only dataset. *Lepiota spiculata* was found in our phylogenetic analyses to be part of *Lepiota* sect. *Ovisporae* (J.E. Lange) Kühner, in the subincarnata/brunneoincarnata clade, which is known to harbor amatoxin-containing species (Vellinga, 2001, 2003; Zhang et al., 2019). Based on this taxonomic placement, we hypothesized that *L. spiculata* might also produce amatoxins and could therefore be considered a poisonous species. A positive outcome of the Wieland-Meixner spot test (Meixner, 1979) on our specimens hinted at the occurrence of hydroxy-substituted indole-containing metabolites in the basidiomes. Subsequent chemical analysis by means of HPLC–MS analysis confirmed unequivocally the presence of amatoxins; in particular, α-amanitin was found to reach levels of 3.9 mg/g dry weight in the pileus, which render *L. spiculata* a potentially lethal mushroom species, if consumed.

## Materials and Methods

### Fungal Material Collection

Specimens examined (one adult and one at the primordium stage) were collected in a hilly forest near the Sosúa cemetery in the Puerto Plata province, Dominican Republic, and are deposited at the Dr. Rafael Ma. Moscoso National Botanical Garden herbarium (JBSD). Institutional herbarium acronyms follow Index Herbariorum (Thiers, 2020), while “ANGE” refers to the personal herbarium of CA.

### Morphological Studies

Macroscopic and microscopic descriptions are based exclusively on the recent collection made in the Dominican Republic. Color terms in capital letters (e.g., ORANGE 226, pl. XVI) are from Séguy (1936). Terminology for descriptive terms is according to Vellinga (1988, 2001). Photographs of collections were taken in the natural habitat using a Nikon Coolpix 8400 digital camera. Microscopic anatomical features were observed and recorded from revived dried material; sections were rehydrated either in water or in 5% potassium hydroxide (KOH). All microscopic structures were observed and measured from preparations in anionic Congo Red. Colors and pigments were described after examination in water. Measurements were made at 1000 × using a calibrated ocular micrometer (Meiji MT4310H optical light microscope). Basidiospores were measured directly from the hymenophore of mature basidiomes. The notation [n/m/p] indicates that measurements were made on “*n*” randomly selected basidiospores from “m” basidiomes of “*p*” collections – Q = length/width ratio, Qm = average quotient (length/width ratio) – and the average spore volume (V) was approximated as a rotation ellipsoid [V = (π.L.W2)/6]. The width of each basidium was measured at the widest part, and the length was measured from the apex (sterigmata excluded) to the basal septum. Metachromatic and iodine reactions were tested by staining the basidiospores in Brilliant cresyl blue and Melzer’s reagent, respectively. Line drawings of microstructures were traced in freehand based on digital photomicrographs of rehydrated material.

### DNA Extraction, PCR Amplification, and DNA Sequencing

Total DNA was extracted from dry specimens employing a modified protocol based on Murray and Thompson (1980). PCR amplification was performed with the primers ITS1F and ITS4 (White et al., 1990; Gardes and Bruns, 1993) for the nrITS region, while LR0R and LR5 (Vilgalys and Hester, 1990; Cubeta et al., 1991) were used to amplify the 28S rDNA region, EF1-983F and EF1-1567R (Rehner and Buckley, 2005) for the translation elongation factor 1a (TEF1) gene, and finally bRPB2-6F and bRPB2-7R2 for the RNA polymerase II second largest subunit (RPB2) gene (Liu et al., 1999; Matheny et al., 2007). PCR reactions were performed under a program consisting of a hot start at 95°C for 5 min, followed by 35 cycles at 94°, 54°, and 72°C (45, 30, and 45 s, respectively) and a final 72°C step for 10 min. PCR products were checked in 1% agarose gels, and positive reactions were sequenced with one or both PCR primers. Chromatograms were checked searching for putative reading errors, and these were corrected.

The sequences were submitted to GenBank^1^, and their accession numbers are reported in Table 1.

**TABLE 1:** Sequences used in the combined phylogenetic analyses. Newly generated sequences are boldfaced.

Species	GenBank acc. number			
	
	nrITS	nrLSU (28S)	*tef1*	*rpb2*
*Agaricus bisporus*	GU327642	FJ755218	–	AF107785
*Amanita brunnescens*	AY789079	AY631902	AY881021	AY780936
*Chlorophyllum rhacodes*	AF482849	AY176345	HM488885	HM488803
*Clarkeinda trachodes*	HM488751	HM488771	–	HM488802
*Coniolepiota spongodes*	HM488756	HM488774	HM488883	HM488796
*Eriocybe chionea*	HM488752	HM488773	–	HM488800
*Lepiota* aff. *pilodes*	EF080865	–	HM488942	HM488816
*Lepiota clypeolaria*	JN944093	JN940281	–	JN993691
*Lepiota cristata*	JN944091	JN940283	KY419048	JN993699
*Lepiota fuscovinacea*	AY176372	AY176373	HM488949	HM488817
*Lepiota maculans*	HM222939	HQ832458	–	HQ832436
*Lepiota magnispora*	JN944088	JN940286	GQ258492	JN993681
*Lepiota spheniscispora*	AF391001	AY176404	HM488951	HM488813
***Lepiota spiculata***	**MK696156**	**MK696155**	**MK696577**	**MK696576**
*Lepiota subincarnata*	KC556779	MK278273	HM488952	HM488815
*Leucoagaricus americanus*	AY176407	AF482891	HM488888	HM488809
*Leucoagaricus rubroconfusus*	KP300875	–	HM488916	HM488853
*Macrolepiota procera*	AY243588	AF482880	HM488892	HM488868
*Melanophyllum haematospermum*	AY176494	AY176456	HM488947	HM488818
*Pluteus* aff. *romellii*	AY854065	AY634279	AY883433	AY786063

### Sequence Alignment, Data Set Assembly, and Phylogenetic Analyses

A four-gene dataset (nrLSU, nrITS, *rpb2*, and *tef1*) was assembled, which includes a wide sampling of the agaricoid genera in the Agaricaceae (Sysouphanthong et al., 2011). All species of *Lepiota* (Pers.) Gray with available *rpb2* data in GenBank were included in the analysis. The final dataset includes 18 species in the Agaricaceae, with *Amanita brunnescens* G.F. Atk. and *Pluteus* aff. *romellii* chosen as outgroup taxa. Sequences used in the analyses are listed in Table 1. Separately, we assembled an nrITS dataset that includes a wide representation of the different lineages of *Lepiota* sensu Vellinga (2003, 2004b), including also species of *Lepiota* sect. *Echinatae* Fayod, *Cystolepiota* Singer, and *Melanophyllum* Velen. The final dataset consists of 243 nrITS sequences, and the midpoint rooting option was chosen to root the tree. All sequences were aligned using MAFFT version 7 (Katoh and Toh, 2008), and the strategy FFT-NS-i was selected. Alignments were inspected and manually corrected using AliView (Larsson, 2014). Maximum likelihood (ML) analyses were run using RAxML version 8.2.10 (Stamatakis, 2014), under a GTR model with one hundred rapid bootstrap (BS) replicates. The ML analyses were run using the resources at the CIPRES Science Gateway (Miller et al., 2010).

### Wieland-Meixner Test

A small fragment of dried *L. spiculata* was rehydrated with water and squeezed to obtain a drop of mushroom juice that was spotted onto a piece of lignin-containing paper and left to evaporate at room temperature. Two drops of concentrated hydrochloric acid (37% w/w) were placed directly onto the resulting spot and left to react at room temperature away from direct sunlight. The appearance of a bluish-green color within a few minutes indicated a positive result for the Wieland-Meixner spot test (Meixner, 1979; Beutler and Vergeer, 1980; Walton, 2018). As the negative control, a drop of concentrated HCl was spotted alone onto the paper matrix.

### Chemical Analysis

Prior to chemical analysis, all samples of *L. spiculata* were further dried at 55°C under airflow for 24 h. Pileus and stipe were analyzed separately. Dried mushroom samples were ground and homogenized in methanol/water/0.01 M HCl (5:4:1 v/v/v) as the extraction solvent using a tissue homogenizer and a 1:30 (w/v) sample-to-solvent ratio. After 24 h extraction, samples were centrifuged for 10 min at 5000 rpm and the supernatant was filtered through a 0.22 μm syringe filter, and 10 μL aliquots of the resulting mushroom extracts were subjected to reversed-phase HPLC analysis. Standards for α-amanitin and phalloidin were obtained from Sigma-Aldrich (United States), whereas β-amanitin, γ-amanitin, and phallacidin were from Enzo Life Sciences (Farmingdale, NY, United States). All solvents used were HPLC grade. Stock solutions of all standards at 100 μg/mL were prepared in methanol. Calibration standards were diluted in the extraction solvent at concentrations of 1, 10, 20, 100, 200, and 1000 ng/mL; for each standard calibration, curves were linear over the range of interest (*r*^2^ > 0.99). Mushroom extracts were analyzed in triplicate; data are given as mg toxin per g dried mushroom (mean ± standard deviation) for each sample.

Separation and quantification of toxins in samples of *L. spiculata* was performed by reversed-phase HPLC analysis following well established procedures (Yilmaz et al., 2013, 2015, 2018). A Shimadzu instrument (Japan) fitted with an RP-C18 column (5 μm particle size; 150 mm × 4.6 mm; Agilent Technologies, Palo Alto, CA, United States) was used and equipped with an online diode-array detector (DAD). Isocratic elution with 0.05 M ammonium acetate (pH 5.5 with acetic acid)/acetonitrile 90:10 (v/v) as the mobile phase was used at a flow rate of 1 mL/min at 40°C. Detection was performed at 303 nm for amatoxins and at 290 nm for phallotoxins, and UV absorption spectra were recorded online with the photodiode-array detection system. Detection limits were determined to be 2.5 ng/g dry weight for amatoxins and 2.8 ng/g dry weight for phallotoxins.

In order to confirm unambiguously the identity of the toxins, mass spectra of the relevant peaks in mushroom extracts were recorded with a Shimadzu (Japan) 8040 MS/MS instrument equipped with an ESI source operating at a spray voltage of 3 kV in the ESI-positive ionization mode, following LC–MS/MS conditions similar to those reported by Gicquel et al. (2014). The capillary temperature was 350°C, and nitrogen was supplied as drying gas with a pressure of 35 p.s.i.g (1 p.s.i. = 6894.76 Pa). Data were acquired in targeted single ion monitoring (SIM) mode and scanning mode simultaneously. Tuning parameters were optimized by direct infusion of individual standards at a concentration of 1 μg/mL in the mobile phase into the ionization probe at a flow rate of 0.4 mL/min in the ESI-positive mode.

## Results

### Taxonomy

***Lepiota spiculata*** Pegler, Kew Bulletin, Additional Series 9: 390, 1983.

(Figures 1–3)

#### Description

**FIGURE 2:**
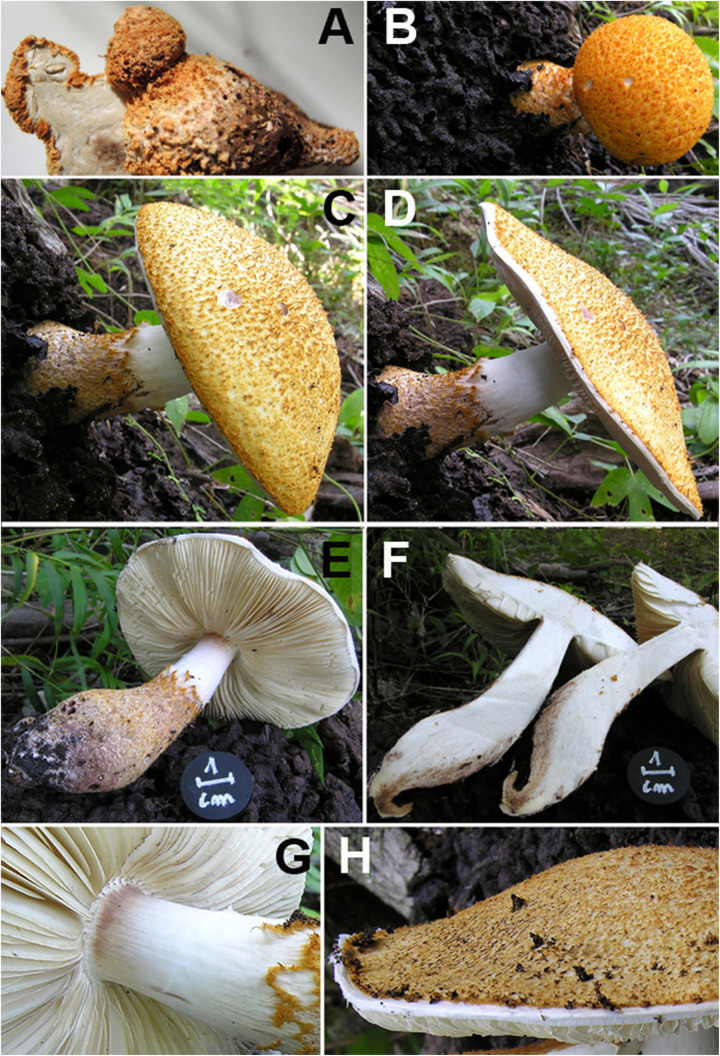
*Lepiota spiculata*. Macroscopic characters. **(A)** Primordium in section. **(B–D)** Pileus in various stages of development. **(E)** Stipe. **(F)** Basidiome in section. **(G)** Lamellae. **(H)** Pileus surface at maturity (photos by C. Angelini).

**FIGURE 3:**
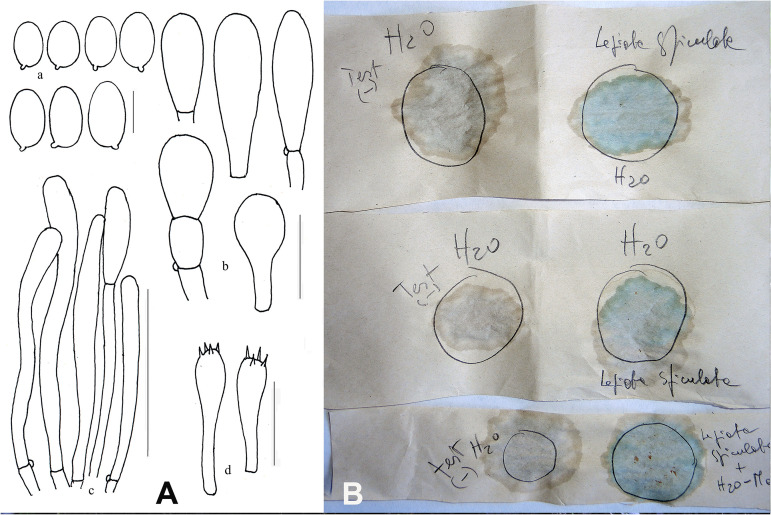
*Lepiota spiculata*. **(A)** Ink drawing of microscopical traits. Bars: (a) spores 5 μm; (b) cheilocystidia 20 μm; (c) pileipellis 100 μm; (d) basidia 20 μm (drawings by A. Bizzi). **(B)** Wieland-Meixner test: negative control (left column) and blue-green positive reaction (right column) (photo by C. Angelini).

*Pileus* 7 cm in diam., spherical-globose, then hemispherical, finally expanding to plano-convex (Figures 2B–D); surface at first formed by a thick brown-ochre (ORANGE 176, Pl. XII) layer of wooly appearance (which covers the whole basidiome in the primordium), then consisting of pyramidal warts with yellow-orange curved apices (ORANGE 196, Pl. XIV) with reddish tips (ORANGE 151, Pl. XI) that completely cover the pileus, at complete maturity ochraceous buff (ORANGE 211, Pl. XV) with the brownish apices (ORANGE 176, Pl.VII) covering above all the central part, while toward the periphery the layer tends to rise up to the margin and then breaks into irregular squamules on a white background (Figure 2H). Margin strongly inrolled in the primordium almost completely curling over the lamellae (Figure 2A). *Lamellae* free, or sub-free-annexed, never distant, white (BLANC ABSOLU, Pl. XLIX), lilac-vinaceous when bruised (ROUGE 11, Pl. I) (Figure 2G), quite thick, ventricose, 6–7.5 mm wide, with the edge slightly eroded, lamellulae of various lengths and usually 1–2 between each pair of lamellae reaching the stipe. *Stipe* 8 × (1–)1.5(–3) cm, cylindrical, attenuated at the apex (1 cm wide), with the base swollen (3 cm wide) and rooting, solid, at the apex white and smooth, more scaly below, ochre-brownish (ORANGE 176, Pl.VII); all parts lilac-vinaceous on bruising (Figure 2E). *Annulus* white, evident only in the primordium, then fragile, fugacious, almost absent, separating the apical glabrous part and the lower scaly part of the stipe. *Context* very thick, up to 1 cm at the disk, white, unchanging on exposure, or lilac-vinaceous when bitten by insects or mollusks (Figure 2F); odor none; taste mild, subfarinaceous. *Basidiospores* [50/1/1] (5.0–) 5.5–8.0 × 3.5–4.5 μm, on average 6.5 ± 0.7 × 4.1 ± 0.2 μm, Q = 1.38–2.00, Qm = 1.60, V = 57 μm^3^, ellipsoid to oblong, thin-walled, hyaline, dextrinoid, not metachromatic, with wall that does not swell in either ammonia or acetic acid. *Basidia* 18–36 × 6–7 μm, cylindrical-clavate, tetrasporic. Lamella edge sterile, with crowded cheilocystidia. *Cheilocystidia* 20.5–34 × 10–13.5 μm, clavate, subfusiform, hyaline, thin-walled. *Pleurocystidia* absent. *Pileus covering* a conspicuous but discontinuous trichoderm of erect hairs, consisting of hyphae 65–175 × 10–22.5 μm wide, gathered in thin needle-like tufts, flexuous, often irregular, with tapered or clavate apices, with thickened walls with yellow-ochre (VERT337, Pl. XXIII) parietal pigment. *Stipitipellis* a cutis consisting of cylindrical, 4–10 μm-wide hyphae, with squamules as on pileus. *Clamp-connections* abundant, especially at the base of the cheilocystidia and on the hyphae of the pileus covering and context (see microscopic drawings in Figure 3A).

#### Material Studied

Dominican Republic, municipality of Sosúa, Puerto Plata province, local cemetery, 19°44′4″N, 70°32’21”E, 50 m a.s.l., two specimens (one adult and one at the primordium stage) growing on an abandoned termite nest, 06 Dec. 2017 – *Leg.* C. Angelini, *Det.* E.C.Vellinga. (JBSD127426, ANGE971) (Figure 1A).

### Phylogenetic Position of the Species

**FIGURE 4:**
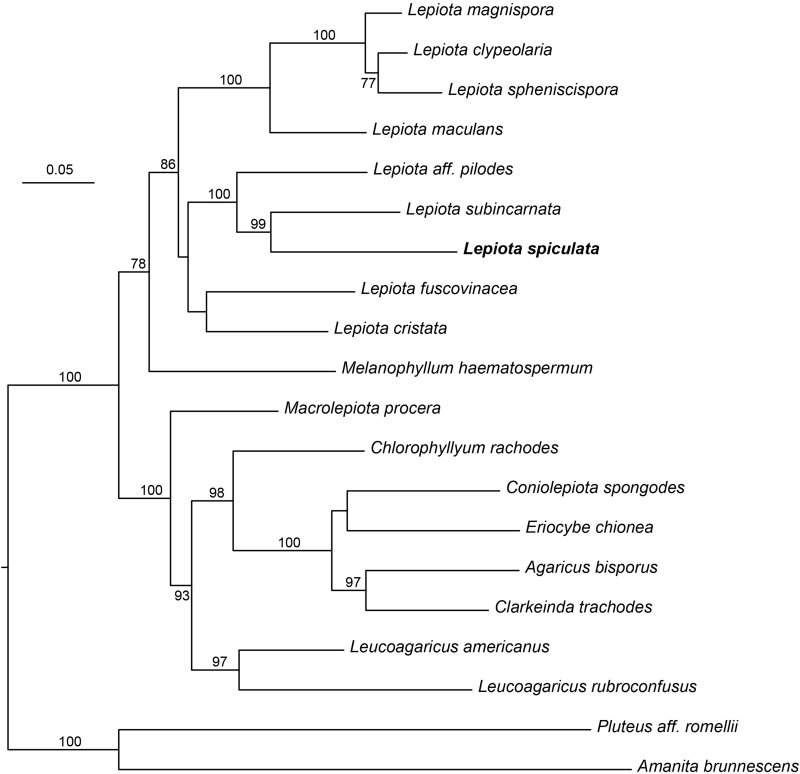
Phylogenetic position of *Lepiota spiculata* based on a Maximum likelihood analysis of a combined nrITS/nrLSU/*tef1*/*rpb2* data set.

In the 4-gene dataset (Figure 4), *L. spiculata* appears as the sister species to *L. subincarnata* J.E. Lange with high support. Both species are part of a more inclusive clade with *Lepiota* aff. *pilodes*. The genus *Lepiota*, sensu Vellinga (2003), receives moderate support in this dataset, either with or without the inclusion of *Melanophyllum* Velen.

**FIGURE 5:**
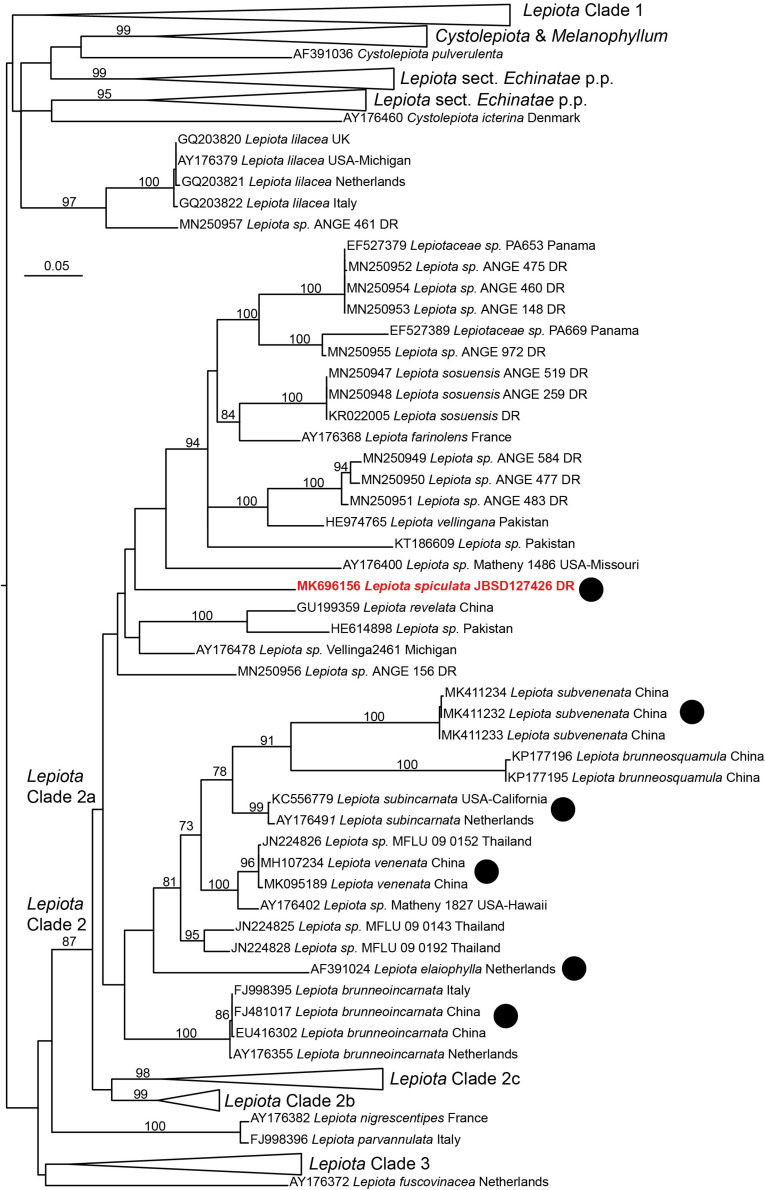
Phylogenetic position of *Lepiota spiculata* based on a maximum likelihood analysis of an nrITS dataset. Clade names follow Vellinga (2003). The black dots indicate taxa with confirmed presence of amatoxins. DR, Dominican Republic.

The nrITS sequence of *Lepiota spiculata* is located in the nrITS analysis as part of the *Lepiota* Clade 2a in the sense of Vellinga (2003), even though this placement does not receive high support in the analyses (Figure 5). This clade includes species traditionally placed in *Lepiota* subsect. *Helveolinae* Bon & Boiffard, i.e., *L. brunneoincarnata* Chodat & C. Martín, *L. subincarnata* J.E. Lange, *L. elaiophylla* Vellinga & Huijser (Vellinga, 2001), all known to contain amatoxins (Vellinga, 2001; Sgambelluri et al., 2014; Walton, 2018). Two amatoxin-containing species recently described from China, *Lepiota venenata* Zhu L. Yang & Z.H. Chen (Cai et al., 2018) and *Lepiota subvenenata* Hai J. Li, Y.Z. Zhang & C.Y. Sun (Zhang et al., 2019), are also placed in Clade 2a (Figure 5). *Lepiota brunneosquamula* J.F. Liang & Z.L. Yang appears as sister to *L. subvenenata* (Figure 5), and while the presence of amatoxins in this species has been suggested due to its phylogenetic placement (Zhang et al., 2019), it has yet to be confirmed by chemical analyses.

### Wieland-Meixner Test

The Wieland-Meixner test on dried specimens of *L. spiculata* resulted in a clear positive reaction, viz., a blue-green color (Figure 3B), thereby leading support to our initial surmise. The Wieland-Meixner spot test is most useful and straightforward to detect the presence of hydroxylated indole derivatives such as, for instance, amatoxins (e.g., α-, β-, γ-, ε-amanitin and amanullin); likewise, however, it also detects 4- and 5-substituted tryptophan derivatives such as psilocin (*N*,*N*-dimethyl-4-hydroxytryptamine), bufotenin (*N*,*N*-dimethyl-5-hydroxytryptamine), serotonin (5-hydroxytryptamine), and 5-hydroxytryptophan (Beutler and Vergeer, 1980; Walton, 2018). In the case of 5-hydroxytryptamines, the positive Wieland-Meixner spot test is preceded by an initial discoloration to reddish-brown colors, whereas 4-hydroxytryptamines are reported to display a preliminary shift to gray hues before discoloring ultimately to blue-green (Beutler and Vergeer, 1980). Since lepiotaceous fungi are not known to produce 4- or 5-hydroxylated tryptophan-derived metabolites, and given the fact that the Wieland-Meixner spot test on *L. spiculata* did not show any preliminary discoloration to either reddish-brown or gray colors, this suggested that the direct reaction to blue-green was likely due to the presence of amatoxins.

### Chemical Analysis

**FIGURE 6:**
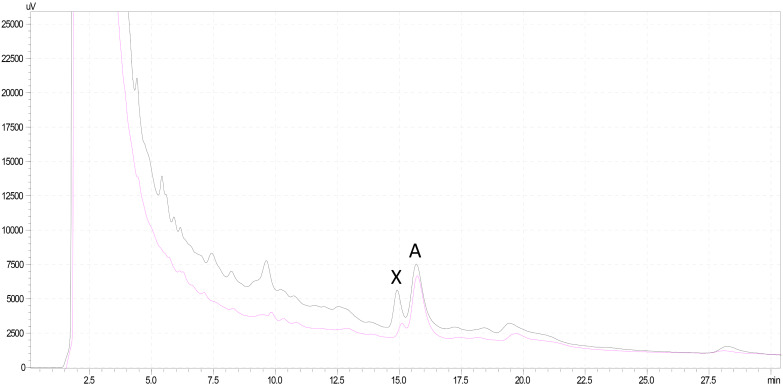
HPLC chromatogram of *Lepiota spiculata* extracts at 290 nm featuring α-amanitin (A) and an unknown peak (X): black line corresponds to pileus extract, pink line to stipe extract; for chromatographic conditions, see text.

**FIGURE 7:**
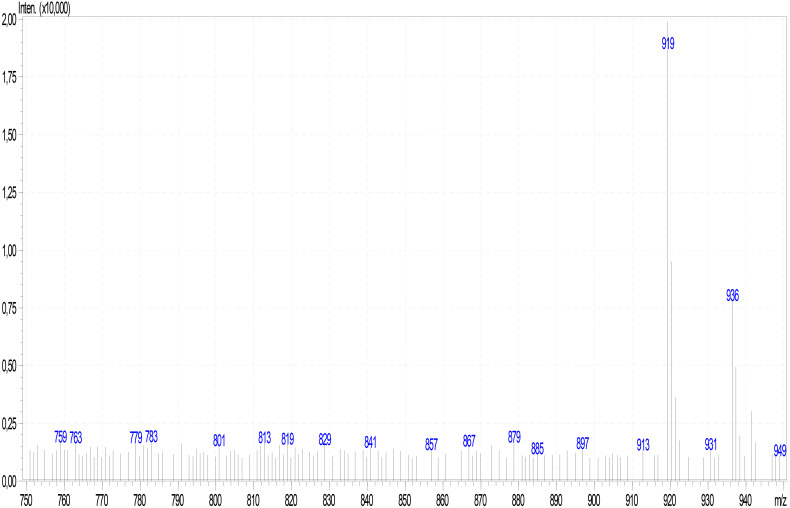
ESI mass spectrum of α-amanitin in *Lepiota spiculata* extracts.

**FIGURE 8:**
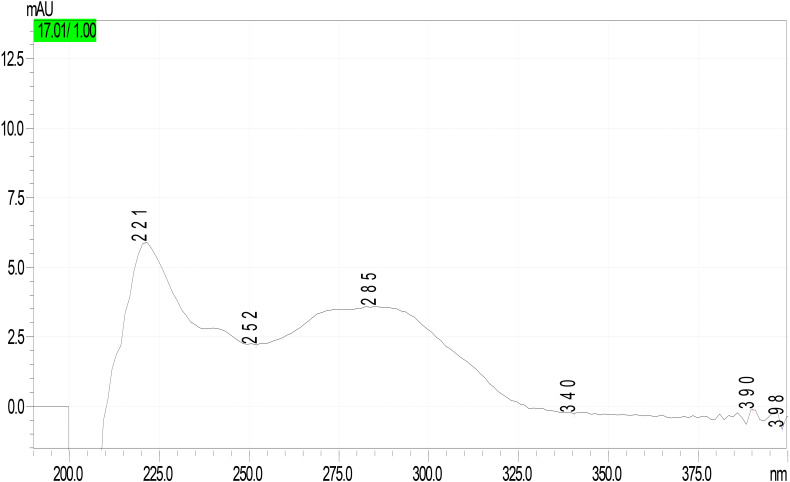
UV-DAD spectrum of unknown peak in *Lepiota spiculata* extracts.

Analysis of mushroom extracts by means of reversed-phase HPLC–UV-DAD and LC–MS/MS confirmed unequivocally that *L. spiculata* contains α-amanitin (Figure 6); the concentration of the toxin is highest in the pileus, where a level of 3.9 mg/g dry weight was measured (Table 2). α-Amanitin gave the expected [M + H]^+^ ion at *m/z* 919.35 by LC–MS/MS ESI analysis in the positive ionization mode (Figure 6). β-Amanitin, γ-amanitin, phalloidin, and phallacidin were not detected in either pileus or stipe (Figure 6). Interestingly, an additional minor peak that did not correspond to any of the toxin standards used was detected in extracts of *L. spiculata* (Figure 7); this peak eluted earlier than α-amanitin under the RP-HPLC conditions employed (*t*_R_ 14.9 vs. 15.7 min, respectively). The UV spectrum for the unknown peak is reported in Figure 8 and resembles a phallotoxin-type spectrum, but in the absence of additional spectroscopic data its identity must remain unanswered at present. By analogy to α-amanitin, the level of the unknown toxin is higher in the pileus of *L. spiculata*, where it reached 1.2 mg/g dry weight (expressed as α-amanitin equivalent) (Table 2).

**TABLE 2:** Toxin levels in *Lepiota spiculata* (in mg/g dry weight) as determined by HPLC analysis.

	α-Amanitin	β-Amanitin	γ-Amanitin	Phalloidin	Phallacidin	Unknown toxin*
Pileus	3.9 ± 0.1	–	–	–	–	1.2 ± 0.1
Stipe	2.7 ± 0.1	–	–	–	–	0.5 ± 0.05

*In the absence of an analytical standard, the concentration of the unknown toxin in L. spiculata is expressed as α-amanitin equivalent.

## Discussion

*Lepiota spiculata* was described by Pegler based on a collection by Fiard in Martinique. The original description includes two *in situ* photographs and one ink drawing from the holotype with macroscopical and microscopical traits (Pegler, 1983). The exsiccatum of the holotype was deposited in Kew (K) along with the drawing by Fiard himself (this drawing no longer exists, either at K, or in LIP herbaria – *personal communications*). Our collection from the Dominican Republic matches exactly with macroscopic and microscopic traits of the original collection from Martinique (Pegler, 1983). The only difference in the Dominican collection is the almost total absence of a true annulus (partial veil) on the stipe (Figure 2G), which is clearly visible only in the section of the primordium (Figure 2A).

Based on the few collections made (Pegler, 1983; Singer and Garcia, 1989; our report), *L. spiculata* appears to be a strictly termitophilic species. However, despite a well-established body of knowledge on the symbiotic relationships between paleotropical fungus-growing termites (Macrotermitineae) and species of the genus *Termitomyces* R. Heim (Agaricales, Lyophyllaceae; Aanen et al., 2007; Nobre et al., 2011; Ye et al., 2019) and other associated fungi (Visser et al., 2011), the relationship between neotropical *L*. *spiculata* and termites, if any, remains unknown and warrants further ecological investigations.

After considering *L*. *spiculata* as a species of dubious position in *Lepiota* at section level because of its medium-sized basidiomes, robustness, and well-developed cheilocystidia, Pegler (1983) placed it doubtfully in *Lepiota* sec. *Ovisporae* because of its ovo-ellipsoid spores and the trichodermic pileipellis with clavate-cylindrical terminal elements. He also recognized affinities with *Lepiota* species of the sect. *Echinatae* Fayod emend. Knudsen [= genus *Echinoderma* (Locq. ex Bon) Bon] due to the presence of pyramidal verrucae on pileus surface. Interestingly, the present molecular phylogenetic analyses (Figures 4, 5) confirm its placement in sect. *Ovisporae*, close to *L*. *subincarnata* J.E. Lange (= *L*. *josserandii* Bon & Boiffard) and *L*. *brunneoincarnata* Chodat & C. Martín. The massive basidiomes of *L. spiculata* are not typically lepiotoid, and it might well be confused with another genus (*Floccularia* Pouzar or *Amanita* Pers.). Because of its pleasant taste and lack of unpleasant odor, *L. spiculata* could be easily mistaken for an edible mushroom with severe health consequences if consumed due to the presence of α-amanitin in toxic concentrations. In fact, α-amanitin represents the major toxin in *L. spiculata*, where levels of approximately 4 mg/g dry weight are reached in the pileus, whereas β-amanitin, γ-amanitin, phalloidin, and phallacidin are absent; an additional phallotoxin-like toxin is also present in conspicuous amounts, especially in the pileus, but no identification has been attempted in the present work. In *Lepiota*, the pileus seems richer in α-amatoxin than the stipe as suggested by our data (Table 2) and previous analyses of *L. brunneoincarnata* (Yilmaz et al., 2015; Sun et al., 2019). Such high levels of α-amanitin in *L. spiculata* are comparable to those reported in the literature for poisonous *Lepiota* species belonging to the same section and clade, such as *L. subincarnata* and *L. brunneoincarnata* (Enjalbert et al., 2002; Sgambelluri et al., 2014; Yilmaz et al., 2015; Walton, 2018). Although most *Lepiota* species of sect. *Ovisporae* (Vellinga, 2001; Liang et al., 2018) are smaller in size and are much less conspicuous and attractive in appearance (Razaq et al., 2013), they do account for fatal cases. In fact, although several *Amanita* species represent the most notorious source of amatoxins and are responsible for most fatal mushroom poisonings worldwide (Enjalbert et al., 2002; Tang et al., 2016; Wei et al., 2017; Diaz, 2018; Walton, 2018), fatal intoxications after ingestion of amatoxin-containing species of *Lepiota* also occur (Sgambelluri et al., 2014; Diaz, 2018). *Lepiota* poisonings have been reported in Europe, America, Asia, and North Africa (Tunisia) (Paydas et al., 1990; Watling, 1991; Ramirez et al., 1993; Khelil et al., 2010; Kervégant et al., 2013; Kose et al., 2015; Cai et al., 2018; Diaz, 2018; Sun et al., 2019), and the most frequently reported fatal cases are due to *L*. *brunneoincarnata* and *L*. *subincarnata* (Roux et al., 2008; Khelil et al., 2010; Mottram et al., 2010; Diaz, 2018; Sun et al., 2019). Lethal *Lepiota* species usually produce more than one type of amatoxin, for example, *L. brunneoincarnata* contains both α- and β-amanitin (Yilmaz et al., 2015), whereas α- and γ-amanitin are present in *L*. *josserandii* (Sgambelluri et al., 2014; Walton, 2018). By contrast, *L*. *spiculata* contains only α-amanitin and *L. venenata* Zhu L. Yang & Z.H. Chen, which has been recently responsible for fatal poisoning cases in China (Cai et al., 2018), has been shown by analysis of genomic data to possess only the genes coding for α-amanitin (Lüli et al., 2019). Species of the genus *Galerina* Earle also produce amatoxins at toxicologically relevant levels and are responsible for human and animal poisonings (Walton, 2018); in particular, wood-rotting basidiomes of *G. marginata* (Batsch) Kühner contain α-, β-, and γ-amanitin at a combined level of 0.7–2.1 mg/g dry weight (Enjalbert et al., 2004; Walton, 2018), whereas α- and β-amanitin have been measured at a combined concentration up to 5.6 mg/g dry weight in Japanese specimens of *G. helvoliceps* (Berk. & M.A. Curtis) Singer (Muraoka et al., 1999).

For the sake of comparison, concentrations of α-amanitin in *L. spiculata* are higher than in *Amanita phalloides* (Fr.) Link, but the latter also contains other clinically relevant amatoxins such as β-amanitin that contribute to the overall amatoxin load (Walton, 2018). With regard to *L. spiculata*, therefore, the α-amanitin levels that have been detected in the basidiomes are toxicologically relevant and render *L. spiculata* a potentially lethal species, if ingested. Hence, based on such data, even in the absence of poisoning reports *L. spiculata* must be listed accordingly among poisonous mushroom species.

It is desirable that the other few *Lepiota* species morphologically and/or molecularly assignable to section *Ovisporae* that have been described or reported from the Antilles (Caribbean area) (Pegler, 1983; Justo et al., 2015) will be investigated for the presence of amanitins.

## Data Availability Statement

The datasets generated for this study can be found in the GenBank, MK696156, MK696155, MK696577, and MK696576.

## Author Contributions

CA collected the species. CA and AB were responsible for the morphological analysis and description of the collection. AJ performed the molecular phylogenetic analyses. EK performed the chemical analysis. CA, AV, and PD planned, organized, and evaluated critically the experimental parts and wrote the manuscript. All authors contributed to the article and approved the submitted version.

## Conflict of Interest

The authors declare that the research was conducted in the absence of any commercial or financial relationships that could be construed as a potential conflict of interest.

## Footnotes

1 http://www.ncbi.nlm.nih.gov/genbank/
